# The power to (detect) change: Can honey bee collected pollen be used to monitor pesticide residues in the landscape?

**DOI:** 10.1371/journal.pone.0309236

**Published:** 2024-09-26

**Authors:** Emily A. Carlson, Andony Melathopoulos, Ramesh Sagili

**Affiliations:** Department of Horticulture, Oregon State University, Corvallis, Oregon, United States of America; University of Leipzig Faculty of Life Sciences: Universitat Leipzig Fakultat fur Lebenswissenschaften, GERMANY

## Abstract

Analysis of trapped honey bee pollen for pesticide residues is the most widely used method of monitoring the amount of pesticide entering colonies and its change over time. In this study, we collected and analyzed pollen from 70 sites across four bee-pollinated crops over two years to characterize the variation in pesticide detection across sites, crops and at different periods during bloom. Hazard Quotient, HQ, is the most common way that pesticide residues are aggregated into a single pesticide hazard value in the current literature. Therefore, change in pesticide hazard (HQ) was quantified in composite pollen samples collected from pollen traps and in pollen color subsamples separated into pollen from the target crop being pollinated and pollen from other plant species. We used our estimates of the variation in HQ to calculate the number of sample location sites needed to detect a 5% annual change in HQ across all crops or within specific crops over a 5-year period. The number of sites required to be sampled varied by crop and year and ranged between 139 and 7194 sites, costing an estimated $129,548 and $3.35 million, respectively. The HQ values detectable for this cost would be 575 and 154. We identified additional factors that complicate the interpretation of the results as a way to evaluate changes in pest management practices at a state level. First, in all but one crop (meadowfoam), the pollen collected from outside the crop honey bee colonies were pollinating comprised a major percentage of the total pollen catch. Moreover, we found that when the overall quantity of pollen from different pollen sources was taken into account, differences in HQ among crops widened. We also found that while HQ estimates remain consistent across the bloom period for some crops, such as cherry, we observed large differences in other crops, notably meadowfoam. Overall, our results suggest the current practice of interpreting pesticides levels in pollen may come with limitations for agencies charged with improving pesticide stewardship due to the high variation associated with HQ values over time and across crops. Despite the limitations of HQ for detecting change in pesticide hazard, there remains a potential for HQ to provide feedback to regulators and scientists on field-realistic pesticide hazard within a landscape.

## Introduction

Reducing the exposure of bees to pesticides is a challenge for regulators and land managers. While pesticides are necessary for protecting crops and have documented benefits in controlling pest populations and increasing food security [1–4], they are also linked to the decline of or a negative impact on pollinating insects [5–11]. In the United States, the risk assessment framework used by the Environmental Protection Agency (EPA) is the first line of defense for reducing pesticide impacts on honey bees [12,13], and specify which pesticide use practices have limited risk to pollinating honey bees during the registration or periodic review of those products [12,13]. These practices are then translated onto instructions on pesticide labels that a pesticide applicator must follow. Although the framework aims to characterize risk under field conditions, there remains a need for post-registration monitoring. This need is urgent because many older pesticides have yet to be evaluated using the EPA’s risk assessment, applicators may be using products in ways not specified by the label, and patterns of pesticide spray-drift may move beyond what is evaluated during registration.

In 2014, the EPA expanded pollinator protections to include both a traditional, regulatory approach [12] and individualized state-level voluntary pollinator protection plans [14,15]. Managed Pollinator Protection Plans (MP3s) are used by 31 states [16] to identify, test, and incentivize best management practices (BMPs) to reduce pesticide exposure to honey bees contracted to pollinate crops. An audit of the MP3 program by EPA noted that there are no robust methods to evaluate the effectiveness of the implementation of BMPs outside of surveys of pesticide applicator intentions [17]. States have looked to pesticide monitoring of bee matrices (pollen, honey, wax, etc) as a way to evaluate their efforts to lower pesticide exposure of honey bee colonies over time. The Association of American Pesticide Control Officials, the national body of state agencies responsible for the implementation of MP3s, report that about one third of states use pesticide monitoring in bee matrices (pollen, honey, wax, etc) as part of their state-level plan [18].

Pesticides hazard to honey bees involved in crop pollination is most frequently estimated from pollen trapped from the corbicula of returning foragers [19]. The detection of pesticides in pollen has long been a tool for researchers to estimate the hazard represented to honey bees in agricultural systems [20–27]. Typically, the aggregate hazard of a colony to a pesticide is estimated by dividing the concentration of each pesticide detected in pollen and by its acute oral toxicity to bees, which is summed into a single, unitless value: Hazard Quotient [28,29] (HQ). While HQ, in various iterations, is the most commonly used metric In literature to condense pesticide residues into a single numerical value [28], there are several reasons to doubt if HQ can adequately represent the hazard to bees from pesticides, including its misalignment with the EPA’s own pesticide risk assessment process [29] and the thresholds of concern associated with HQ values with an unclear connection to hive health metrics or survival [30–32]. Moreover, research into the HQ of chemical residues in trapped pollen from honey bees has suggested other limitations associated with relying on this technique for post-registration monitoring of pesticides.

First, honey bees are generalist pollinators which can forage over large scales [33,34] with preferences for the flowers of different plant species [35]. The heterogeneous nature of agricultural landscapes and mix of floral attractiveness within it leads to a situation where pesticide exposure is not always driven by the applications to the crop that honey bees are contracted to pollinate [25,26,36,37]. The fact that not all pollen originates from the pollinated crop is of significance as both pesticide labels and MP3s focus on pesticide stewardship for specific crops. Second, the mass of pollen collected across cropping systems can be very different, in some cases a 43-fold difference [38]. The difference in pesticide residue concentration within the pollen of each floral source [20] can impact the total pesticide exposure of the colony in ways HQ calculations were not designed for [28]. If, for instance, there are two pollen types which present different quantities of pollen and different HQ values to a hive, there currently is not a way to assess the relative risk of these two types of contamination to the health outcomes of the colony [29]. Consequently, while attempts to determine the HQ associated with a specific crop species by sorting the collected pollen to species may provide specific information for pesticide use on the crop [20,24,36], it may over or underestimate the total exposure a colony is experiencing.

In this study, we estimate the variation in pesticide HQ from pollen trapped from crops pollinated by honey bees in the state of Oregon. We used these estimates to determine the power of a post-registration sampling program to detect moderate changes in HQ (5%) over a five-year period, which is reflective of changes of ±1% in toxic load throughout the Pacific Northwest over the past 15 years [39]. We also estimated the costs required to provide sampling with sufficient power to detect these changes. In doing so, we provide insight into using this method as a post-registration monitoring tool and to state-level programs that are using pesticide detection in pollen to evaluate their progress on their MP3 goals. Moreover, by measuring the weight of pollen entering colonies and comparing it to whether it originated from the crop being pollinated or from other plant species, we were able to evaluate the extent to which such sampling could be used to make inferences back to pesticide stewardship on specific crops. Since most sampling programs only sample for a single period during bloom, we also evaluated variation in HQ across three periods of bloom to determine the adequacy of single points of sampling in estimating pesticide hazard over the course of a pollination. Through these efforts we provide guidance to states embarking on programs of post-registration of monitoring of pesticide in pollen collected by honey bees.

## Materials and methods

### Location and pollen trapping

**Fig 1:**
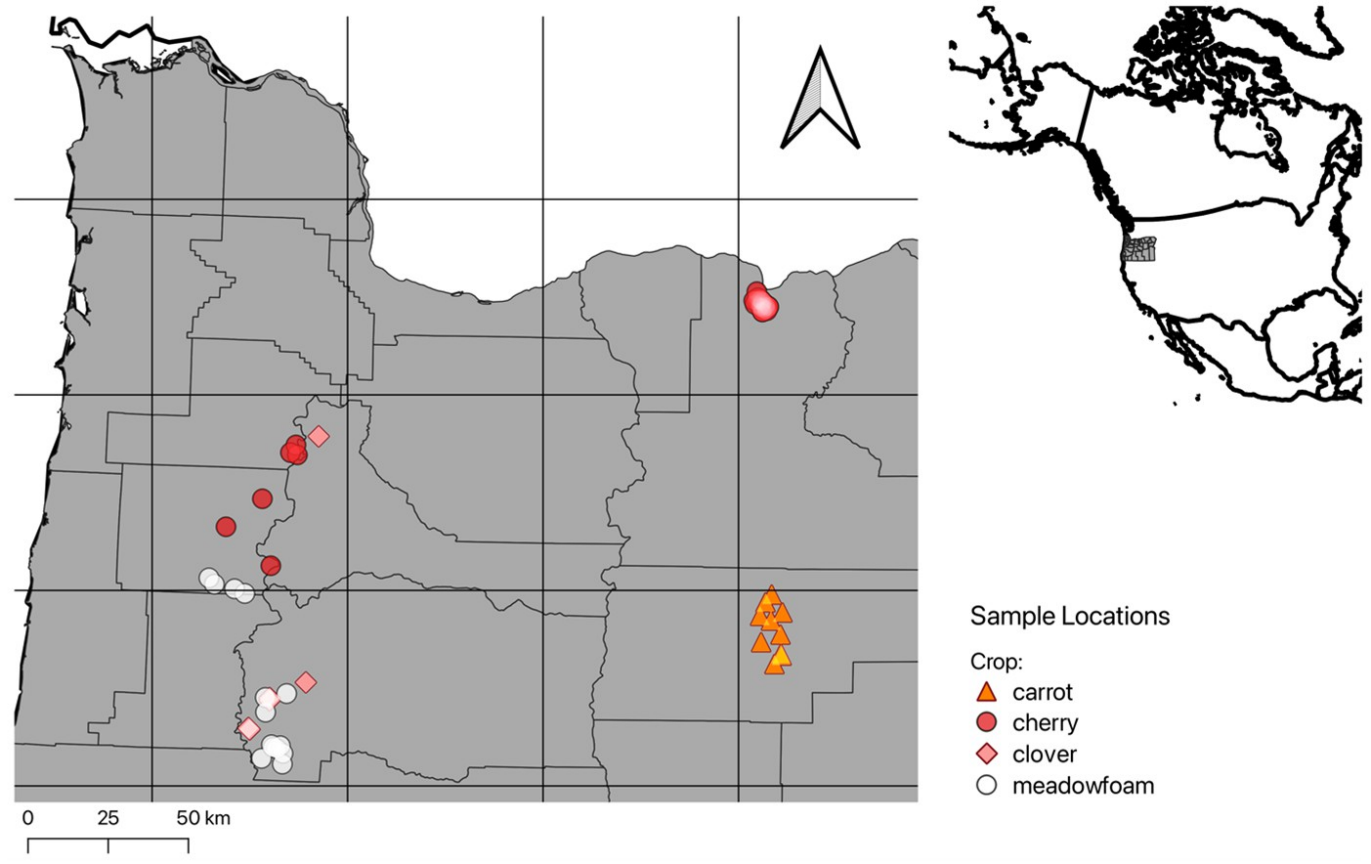
A map showing the location of sites where pollen was trapped from commercial honey bee colonies engaged in pollination contracts throughout Oregon.

This study was conducted over two years (n = 30 sites in 2020, n = 40 sites in 2021) on four pollinator dependent crops in the region (meadowfoam, white clover, sweet cherry and hybrid carrot seed). Commercial meadowfoam (*Limnanthes alba* Hartw. ex Benth., n = 14) and white clover seed (*Trifolium repens* L., n = 6) are produced in the Willamette Valley region of Oregon, USA (Fig 1). The Willamette Valley has productive soils and a temperate climate with a mean annual rainfall of 96–152 cm and was historically an oak savannah and tall grassland habitat with extensive wetlands; today it is characterized by mixed agricultural production, primarily seed crops, nurseries, forests, livestock pasture, and berries [40,41]. Sweet cherry (*Prunus avium* L., n = 12) is grown in the Willamette Valley and in the Columbia Plateau region; the Columbia Plateau is characterized by arid sage-brush steppe and grasslands [40,41]. The area is now agriculturally dominated by sweet cherry, pear, and apple production. Carrot seed production (*Daucus carota* L., n = 9) occurs in the Blue Mountains ecoregion, which was historically desert-like shrubland and grasslands [40,41]; today it is characterized by vegetable seed production, alfalfa, potatoes, and sugar beets. Across all sites, fields averaged 18.9 ha in size (max = 107 ha, min = 5 ha) and were located at least 2 km apart when possible; during bloom, fifteen sites were co-located within 2km of each other (cherry, *n* = 10; carrot, *n* = 0; meadowfoam, n = 3; clover, n = 2). Therefore, there is potential for overlap of the foraging radius of honey bee hives. None of the growers used organic farming techniques, but instead relied on standard agrochemicals and agronomic practices. To perform the field work no permits were required as the colonies were privately held, and we secured permission from each commercial beekeeper to enter the site and collect pollen from their hives.

Pollen trapping was conducted as in Topitzhofer et al., 2021; at each experimental site front porch pollen traps were installed on four honey bee hives on a pallet with strong foraging activity, and all alternative entrances to the hive were closed with tape and foam. After an acclimation period, pollen traps were engaged for at least 24 hr but no more than 48 hr during favorable weather (i.e. periods of time without rain or temperatures below 12.8°C). The total pollen samples were then collected and combined from all four hives and transported in coolers with ice and stored in a -20°C freezer until analysis. For each site, pollen was collected between two and five times throughout the bloom period of the crop (n = 78 pollen trapping events in 2020, n = 132 in 2021). If traps failed due to a leaky pollen trap (i.e. bees could evade the pollen trap), then the trapping event was discarded as a “leak” rather than a true zero where no pollen was collected.

### Pollen acetolysis and identification

The total pollen samples were separated into color groups using the Pantone Color Guide from a 10 g subsample of the whole [19,20]. Each color group was then acetolyzed with a modified protocol for 0.25 g samples [19,42] which removes the lipid coat and allows identification of the pollen protein exine [43,44]. Pollen grains were then identified as focal crop or non-focal crop pollen with light microscopy using DiscoverLife keys (http://www.discoverlife.org) and then confirmed with PalDat Palynological Database (https://www.paldat.org) and Cornell Pollen Grains Reference Library (https://blogs.cornell.edu/pollengrains). Identification was performed to genus and identified as pollen from the focal crop or pollen that originated from outside the focal crop.

### Pesticide residue analysis

Each pollen sample, both the whole sample (composite) and the segregated color groups (color subsamples identified as crop or non-crop), were weighed and placed in 50 mL conical vials with corbicula trapped pollen (1 ≤ 3 g). Pesticide residue analysis was performed by Synergistic Pesticide Laboratory in Portland, Oregon. Composite and color subsamples were shipped overnight in cold storage.

Identity and concentration of pesticide residues were determined through QuEChERS protocol [45] using both LC/MS-MS and GC/MS methods for pollen analysis [10]. Pesticide residues were identified as insecticides, fungicides, or herbicides. Full methodology for pesticide residue analysis is available in (S1 File, pesticide residue testing methodology; S5 File, reporting limits and batch-specific levels of detection for the 292 analytes; and S6 File, pesticide test information including MS polarity).

Hazard Quotient (HQ) was calculated for all samples. HQ is a unitless value which relates the residue detections of a pesticide in bee matrices to the toxicity of that pesticide to individual honey bees, and is used to understand aggregate pesticide entering the colony [28,29]. HQ was calculated for each sample by taking the pesticide residues detected (ppb) and then dividing this value by the LD_50_ of the pesticide. The LD_50_ values used for each pesticide were taken from either Traynor et al (2016) supplementary table or the EPA EcoTOX database (https://cfpub.epa.gov/ecotox/) using the oral LD_50_ for the pesticide to approximate dietary pesticide toxicity. Pesticide label use information was retrieved from the Crop Data Management Systems Database (https://www.cdms.net/Label-Database/Advanced-Search) to determine if the active ingredient was included on an approved label for the crop (note: this database does not include Special Local Need).

Finally, we created a variable which captures the weighted HQ values for different pollen identities entering the colony. For example, HQ > 1000 commonly is used to indicate elevated pesticide levels [28]. We wanted to understand if HQ over 1000 indicates that the same amount of pesticide is entering the colony within each crop. Rather than examining the pesticide hazard through pollen, the Index of HQ weightedness asks if HQ of similar values in each pollen identity provide the same pesticide intake to the colony.

$$\frac{\mu g_{pesticide}}{1g_{pollen}}*g_{pollen\;sample}=\frac{\mu g_{pesticide}}{1\;colony}*\frac{1\;colony}{50,000\;bees}=\frac{\mu g_{pesticide}}{bee}*\frac{1}{LD_{50}}=\%\;LD_{50}\;pesticide$$

$$\%LD_{50\;\left(x\right)}+\%LD_{50\;\left(y\right)}+\;\%LD_{50\;\left(\ldots n\right)}=\text{Index}\;\text{of}\;\text{HQ}\;\text{weightedness}\;\text{to}\;\text{colony}$$

While incoming pollen weight ought to influence the exposure pattern of the colony to a pesticide of a given HQ, it only crudely estimates exposure as pollen consumption is mediated through adult nurse bees [46] and larval consumption rates [29]. Our aim, rather, was to assess the extent to very high or low pollen intake might skew the interpretation of HQ as a proxy for risk.

### Statistical analysis

All statistical calculations and visualizations were performed in the R statistical environment [47] and the specific code we used to analyze this data is available in the (S4 File). Instead of a statistical analysis of HQ across systems, we present an exploratory discussion of the differences between HQ and weightedness of HQ. Comparisons between the mass of pollen sources (i.e. crop and non-crop pollen from the same system) were performed using Welch’s two-sample t-test and ANOVA was used to determine if there was a difference between mean masses of color-sorted samples from each crop system.

We conducted a power analysis on log-transformed HQ (log(HQ+1)) [48] to determine the number of sites needed to detect changes in HQ over time. Log-transformation of the data was necessary to meet the assumptions of normality for the rearranged t-test formula. First, the mean HQ value was calculated for each crop for each year of the study (x_current_). HQ was transformed to normalize the data for use in the power analysis [26,49]. Next, we calculated an annual 5% change in HQ value over 5 years (x_future_) from the untransformed data. This threshold is based on historic change over time; from 1997 to 2012, oral toxic load—defined as the product of the application rate of pesticide and the corresponding acute toxicity to honey bees—increased by 4-fold in one county within the Pacific Northwest, but largely remained unchanged throughout the 15-year time period [39]. We reasoned that a 5% increase or decrease would reflect the worst and best case, respectively, observed historically for our region. We assumed the uniform variance in HQ across all years to compute a pooled standard deviation (sd_pooled_) [50]. The HQ detectable difference was then log-transformed to put it on the same scale as the pooled standard deviation [51]. This allowed us to determine how many sites were needed to detect differences between two sampling periods and what the minimum detectable HQ value difference would be over the sampling scheme. We avoided pseudo-replication of composite samples by performing separate power analysis separated by year (2020 and 2021) and cropping system, considering peak bloom only. Using these parameters we calculated Cohen’s *d* as:

$$d=({\text{x}}_{\text{current}}-{\text{x}}_{\text{future}})/{\text{sd}}_{\text{pooled}}$$

Cohen’s *d* was used to conduct power analyses in R with the package pwr2 using a two-sample power analysis where α = 0.05 and β = 0.2 [48,52,53].

To determine if time significantly influenced the HQ values, we used the complete dataset in a zero-inflated linear mixed model (R packages lme4, caret, and glmmTMB) on the square root transformed HQ values to meet the needs for normal residuals. This model allows for a combination of both a linear relationship between the continuous model of HQ and the binary model where pesticides are or are not detected [54]. The binary model accounts for those factors which influence detection or non-detection of HQ and the GLMM models the HQ as a continuous variable. Next, we created four different ways to test the concept of time as factors. First, we used bloom period (the bloom state of the crop–early, peak, or late). Next, we generated variables for numeric day of the year (1–365), month (01–12) and year (2020 or 2021). Then, we used AICc testing to determine which variables to include in the final model [55,56] and the deviance residuals were used to determine if the final model was appropriate.

### Cost analysis

We calculated cost using a simplified pollen sampling protocol estimating the hours needed to collect and test each composite sample. Travel time to each site was fixed at 2 hours travel (round trip, per visit). Each site requires 3 trips; 1) affix the traps and allows the bees to adjust, 2) engage the traps, 3) collect pollen, release, and remove the traps. Field work on site was estimated to be 1 hour per site, per event. Therefore, the travel and work time for each composite sample obtained is 9 working hours per sample. Each sample also takes between 1–2 hours to process, depending on size. This includes weighing the sample, cleaning it, recording the data, packaging it for transport, and cleaning the traps. We estimate a single sample represents about 10–11 hours of work to collect and prepare. If each pesticide residue panel costs $130 to perform and a qualified field biologist would require $32/hr in wages: $466 is the final cost for collecting, processing, and testing each composite sample. Note that this assumes there is no training required for the field and lab work. Finally, the number of tests needed is twice the number of sites to be tested (2*n*) because each site needs to be tested in year zero (baseline) and five years later to determine a change.

## Results

Over two years, 493 pollen samples were analyzed for pesticide residues for composite (n = 149) and color sorted sub (n = 344) samples. Leaky pollen traps and low pollen collection resulted in the loss of approximately 20 composite samples. Pesticides were detected in 75% of all pollen samples (n = 371) and 87% of composite samples (n = 129). When pesticides were detected, they often resulted in high HQ values, exceeding HQ > 1000 [21,31]. In our study, 30.2% of all contaminated samples (n = 120) and 24% of contaminated composite samples (n = 36) exceeded HQ 1000. Nine total samples and one composite sample exceeded HQ 10,000. Each pesticide detected, the number of times it was detected, and the pollen source are displayed in Table 1.

**Table 1:** A table displaying the pesticides detected in each year by cropping system and pollen type. This includes the number of times that the pesticide was detected within a system, the average residue associated with those detections, and whether the pesticide is approved for use on that crop (excludes Special Local Need).

Year	Crop	Pollen Source	Pesticide	Detection Count	Mean Detection (ppm)	Approved for use in crop
**2020**	**carrot**	**composite**	Dimethenamid	1	0.049	No
			Diuron	1	0.087	
			Fenpropathrin	2	0.241	
			Fenpyroximate	1	0.019	
			Myclobutanil	6	0.032	
			Prometryne	1	0.013	
			Etoxazole	11	0.163	Yes
			Linuron	2	0.087	
			Oxyfluorfen	1	0.156	
			Pendimethalin	13	0.081	
			Sulfoxaflor	2	0.012	
		**crop**	Myclobutanil	1	0.037	No
			Etoxazole	3	0.076	Yes
			Linuron	1	0.057	
			Pendimethalin	2	0.121	
		**other pollen types**	Dimethenamid	2	0.075	No
			Diuron	1	0.127	
			Fenpyroximate	1	0.029	
			Myclobutanil	6	0.047	
			Prometryne	1	0.008	
			Azoxystrobin	1	0.007	Yes
			Etoxazole	15	0.153	
			Linuron	5	0.108	
			Pendimethalin	14	0.107	
			Sulfoxaflor	1	0.021	
**2020**	**cherry**	**composite**	Acequinocyl	2	0.037	No
			Amitraz	18	0.224	
			Carbendazim	1	0.094	
			Diuron	2	0.066	
			Fenpropathrin	1	0.085	
			Fenpyroximate	8	0.021	
			Iprodione	1	0.065	
			Pendimethalin	6	0.045	
			Pronamide	2	0.049	
			Simazine	1	0.030	
			Boscalid	1	0.036	Yes
			Diazinon	1	0.08	
			Difenoconazole	2	0.035	
			Fenbuconazole	3	0.032	
			Fluopyram	11	0.023	
			Penthiopyrad	5	0.342	
			Propiconazole	3	0.097	
			Pyridaben	24	0.058	
			Pyriproxyfen	27	0.340	
			Spirodiclofen	3	0.214	
			Trifloxystrobin	5	0.023	
			Triflumizole	4	0.174	
**2020**	**cherry**	**crop**	Acequinocyl	3	0.018	No
			Amitraz	14	0.080	
			Bifenthrin	1	0.040	
			Carbendazim	1	0.103	
			Dimethenamid	1	0.020	
			Diuron	3	0.080	
			Fenpyroximate	1	0.019	
			Iprodione	5	0.202	
			Pendimethalin	7	0.050	
			Pronamide	2	0.017	
			Simazine	2	0.044	
			tau.Fluvalinate	1	0.017	
			Azoxystrobin	1	0.011	Yes
			Boscalid	3	0.021	
			Captan	2	0.718	
			Difenoconazole	2	0.045	
			Fenbuconazole	2	0.074	
			Fluopyram	7	0.034	
			Penthiopyrad	4	0.285	
			Propiconazole	3	0.176	
			Pyridaben	20	0.079	
			Pyriproxyfen	26	0.283	
			Spirodiclofen	6	0.146	
			Trifloxystrobin	4	0.022	
			Triflumizole	3	0.060	
**2020**	**cherry**	**Other pollen types**	Acequinocyl	1	0.015	No
			Amitraz	4	0.090	
			Forchlorfenuron	1	0.020	
			Iprodione	1	0.107	
			Metalaxyl	1	0.019	
			Pendimethalin	4	0.048	
			Pronamide	1	0.017	
			Simazine	1	0.085	
			Azoxystrobin	1	0.047	Yes
			Boscalid	1	0.026	
			Captan	1	1.560	
			Chlorothalonil	1	0.037	
			Difenoconazole	3	0.041	
			Fenbuconazole	2	0.083	
			Fluopyram	6	0.023	
			Imidacloprid	1	0.014	
			Penthiopyrad	6	0.195	
			Propiconazole	1	0.084	
			Pyridaben	23	0.048	
			Pyriproxyfen	25	0.677	
			Spirodiclofen	4	0.234	
			Trifloxystrobin	3	0.025	
**2020**	**clover**	**composite**	Fenbuconazole	1	0.239	No
			Propiconazole	2	0.025	
			Simazine	1	0.033	
			Azoxystrobin	6	0.021	
			Bifenthrin	4	0.084	Yes
			Flupyradifurone	1	0.569	
			Malathion	3	0.051	
			Pendimethalin	1	0.042	
		**crop**	Boscalid	1	0.010	No
			Azoxystrobin	2	0.015	Yes
			Bifenthrin	2	0.074	
			Flupyradifurone	1	0.008	
			Malathion	1	0.111	
		**Other pollen types**	Boscalid	2	0.027	No
			Fenbuconazole	1	0.875	
			Azoxystrobin	1	0.062	Yes
			Bifenthrin	7	0.119	
			Cypermethrin	1	0.101	
			Malathion	3	0.066	
	**meadowfoam**	**composite**	Bifenthrin	1	0.283	No
			Azoxystrobin	6	0.154	Yes
		**crop**	Bifenthrin	3	0.273	No
			lambda.Cyhalothrin	3	0.274	
			Azoxystrobin	11	0.647	Yes
		**Other pollen types**	Bifenthrin	1	0.047	No
			lambda.Cyhalothrin	1	0.080	
			Azoxystrobin	4	0.179	Yes
2021	**carrot**	**composite**	Etoxazole	3	0.019	Yes
			Metribuzin	1	0.068	
			Pendimethalin	2	0.036	
			Propargite	1	0.163	
			Sulfoxaflor	1	0.009	
	**cherry**	**composite**	Amitraz	1	0.046	No
			Carbendazim	17	0.053	
			Clothianidin	1	0.016	
			Coumaphos	1	0.022	
			Fenpyroximate	2	0.013	
			Flupyradifurone	1	0.014	
			Flutianil	5	0.054	
			Forchlorfenuron	2	0.018	
			Hexazinone	4	0.042	
			Metrafenone	1	0.055	
			Pendimethalin	1	0.019	
			Simazine	1	0.033	
			Spirotetramat	1	0.045	
			Tolfenpyrad	7	0.067	
			Azoxystrobin	2	0.016	Yes
			Boscalid	1	7.630	
			Buprofezin	2	0.012	
			Carbaryl	1	0.018	
			Chlorantraniliprole	8	0.103	
			Fenbuconazole	1	0.515	
			Fluopyram	20	0.035	
			Fluxapyroxad	3	0.024	
			Imidacloprid	9	0.019	
			Malathion	1	0.066	
			Methoxyfenozide	10	0.018	
			Myclobutanil	1	2.080	
			Penthiopyrad	9	0.086	
			Pyraclostrobin	3	1.117	
			Pyridaben	9	0.032	
			Pyriproxyfen	15	0.308	
			Tebuconazole	16	3.180	
			Trifloxystrobin	16	0.038	
			Triflumizole	4	0.056	
2021	**cherry**	**crop**	Amitraz	2	0.043	No
			Forchlorfenuron	2	0.021	
			Hexazinone	2	0.021	
			Simazine	2	0.088	
			Tolfenpyrad	4	0.265	
			Azoxystrobin	1	0.018	
			Fenbuconazole	1	0.095	
			Fluopyram	4	0.218	
			Penthiopyrad	2	0.011	
			Pyridaben	2	0.137	
			Pyriproxyfen	4	0.051	
			Tebuconazole	9	0.866	
			Trifloxystrobin	6	0.116	
		**Other pollen types**	Acetamiprid	1	0.027	No
			Carbendazim	36	0.056	
			Fenpyroximate	1	0.016	
			Flutianil	6	0.053	
			Hexazinone	1	0.020	
			Metrafenone	3	0.087	
			Pendimethalin	1	0.021	
			Simazine	2	0.023	
			Spirotetramat	3	0.014	
			Tolfenpyrad	5	0.236	
			Azoxystrobin	1	0.018	Yes
			Chlorantraniliprole	3	0.023	
			Fluopyram	28	0.056	
			Fluxapyroxad	10	0.0266	
			Imidacloprid	18	0.027	
			Methoxyfenozide	11	0.022	
			Penthiopyrad	14	0.111	
			Pyraclostrobin	2	0.029	
			Pyridaben	4	0.118	
			Pyriproxyfen	5	0.033	
			Tebuconazole	12	0.335	
			Trifloxystrobin	21	0.056	
			Triflumizole	9	0.060	
2021	**clover**	**composite**	Boscalid	1	0.021	No
			Azoxystrobin	8	0.187	Yes
			Bifenthrin	8	0.053	
			Malathion	1	0.083	
		**crop**	Azoxystrobin	9	0.095	Yes
			Bifenthrin	10	0.057	
		**Other pollen types**	Metrafenone	1	0.018	No
			Azoxystrobin	5	0.073	Yes
			Bifenthrin	6	0.084	
	**meadowfoam**	**composite**	Bifenthrin	5	0.045	No
			Buprofezin	1	0.012	
			Clothianidin	1	0.024	
			Fenbuconazole	1	0.052	
			Simazine	1	0.021	
			Azoxystrobin	9	0.217	Yes
			Boscalid	1	0.656	
			Pendimethalin	1	0.213	
			Pyraclostrobin	2	0.156	
		**crop**	Bifenthrin	6	0.125	No
			Simazine	1	0.012	
			Azoxystrobin	12	0.398	Yes
			Propiconazole	1	0.083	
			Pyraclostrobin	1	0.564	
		**Other pollen types**	Bifenthrin	2	0.064	No
			Fenhexamid	1	0.111	
			Azoxystrobin	5	0.179	Yes
			Propiconazole	1	0.024	

Pesticides approved for use on the focal crop and pesticides which do not have a registered label use within the focal crop were both found in pollen. Although this provides some insight into pesticide hazard within these floral resources, caution should be used when connecting these detections to spray habits of landowners. Rather, this illustrates the pervasiveness of pesticides in agricultural settings [10,57–59]. Both approved and unapproved active ingredients were detected in all pollen samples (Table 1). By sub-sampling the pollen into color groups, pesticides were also detected in color-sorted sub samples that were not detected in the composite sample.

First, we estimated that the number of sites that would need to be sampled to detect a 5% change in HQ, and these varied considerably by crop and across year of sampling (Table 2). For example, there was a 22-fold difference between the minimum number of sites and test between the crop with the smallest (cherry) and largest (meadowfoam) required number of tests to detect changes in HQ in 2020. Whereas the difference was 13-fold for composite samples collected in 2020 and 2021. In general, estimating HQ without respect to crop required a median number of tests. Based on our estimates of labor costs, detecting a 5% difference in meadowfoam and clover were the most expensive to conduct, whereas cherry was the least expensive to sample. Carrot samples were too few to calculate *n* in 2021.

**Table 2:** A table displaying the results of the power analysis (n) and cost analysis in USD of sampling schemes. The value *n* represents the number of sites needed for each year that sampling occurs; the number of tests represents 2*n*^*a*^, the required sampling effort for both sampling years. The results show how many composite samples would need to be taken to detect a 5% change in HQ values, based on log-transformed data.

Sampling Scheme	Year	*n* Sites	Number of tests ^a^	Cost (USD)	HQ difference detectable
Cherry	2020	139	278	$129,548	575
Carrot		705	1410	$657,060	8
Clover		370	740	$344,840	0.2
Meadowfoam		3112	6224	$2,900,384	259
Any crop, all peak		2586	5172	$2,410,152	210
Cherry	2021	248	496	$231,136	210
Carrot					
Clover		190	380	$177,080	394
Meadowfoam		3597	7194	$3,352,404	154
Any crop, all peak		188	376	$175,216	149

^*a*^–The number of tests was twice the number of sites, to reflect that the sampling was performed across two different years.

**Fig 2:**
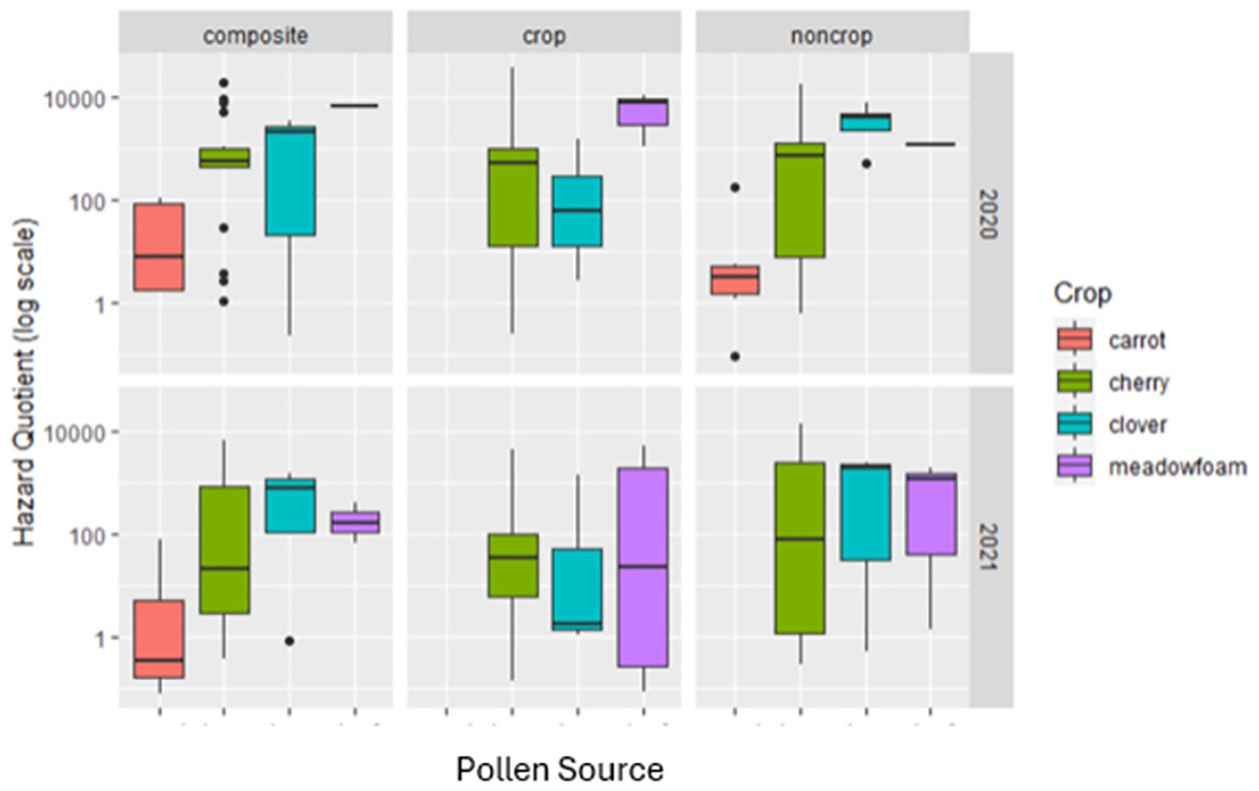
A box and whisker plot of the HQ value of each pollen source within composite and color-sorted subsample. The horizontal line indicates the median value (HQ); the boundaries of the box are the 25^th^ and 75^th^ percentiles. The whiskers represent the most extreme datapoints that are no more than 1.5 times the length of the box.

We found evidence that HQ values were not consistent across crops in composite samples with cherry registering twice the HQ estimate compared to meadowfoam during peak bloom in 2020. However, this trend did not hold for 2021 composite samples (Fig 2).

**Fig 3:**
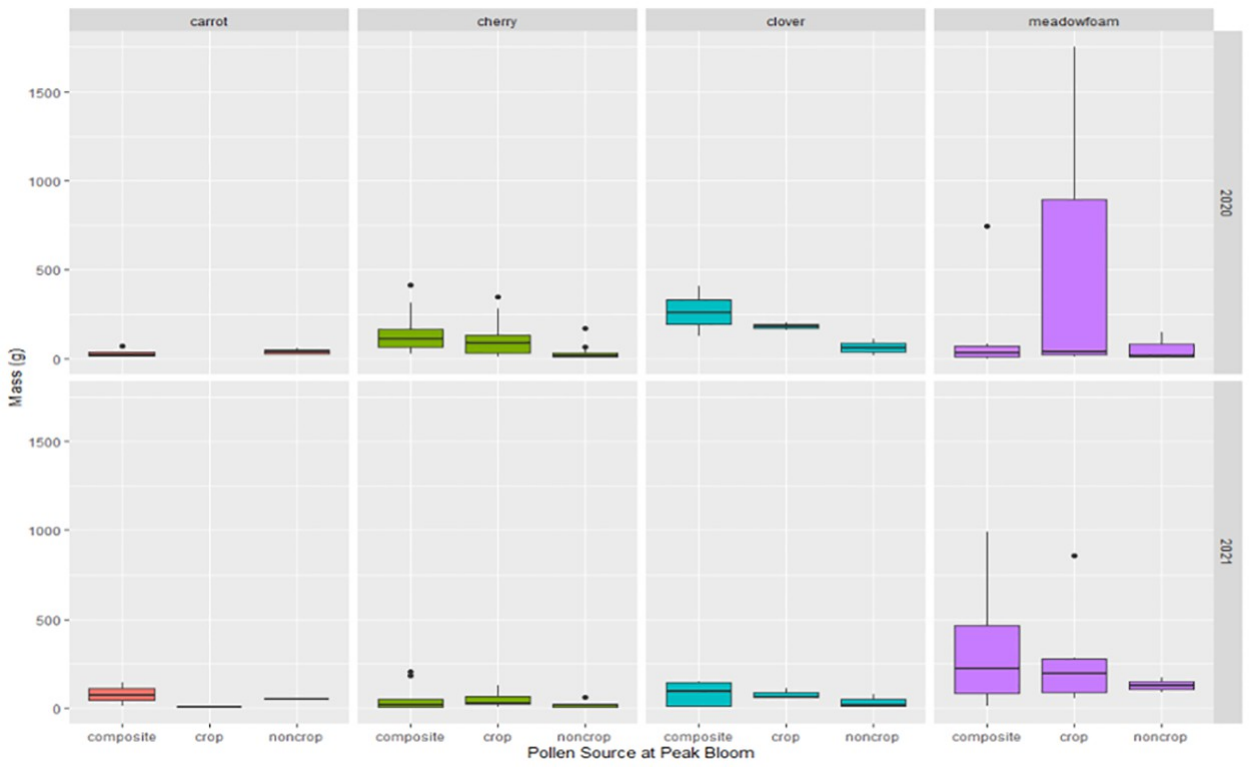
A box and whisker plot showing the mass of pollen collected at peak bloom by pollen identity. The horizontal line indicates the median value (mass); the boundaries of the box are the 25^th^ and 75^th^ percentiles. The whiskers represent the most extreme datapoints that are no more than 1.5 times the length of the box.

The mass of pollen collected by honey bees at peak bloom varied by crop at peak bloom (Fig 3). Differences occurred both among the total pollen collected, with the amount of crop pollen collected. The total amount of pollen collected from meadowfoam was 4.6 and 4.3 times the amount of pollen collected from carrot seed in 2021 and 2022, respectively. The mass of collected from three systems were dominated by the focal crop pollen, with the exception of carrot seed, where it constituted a minority of the sample, if it was collected at all.

**Fig 4:**
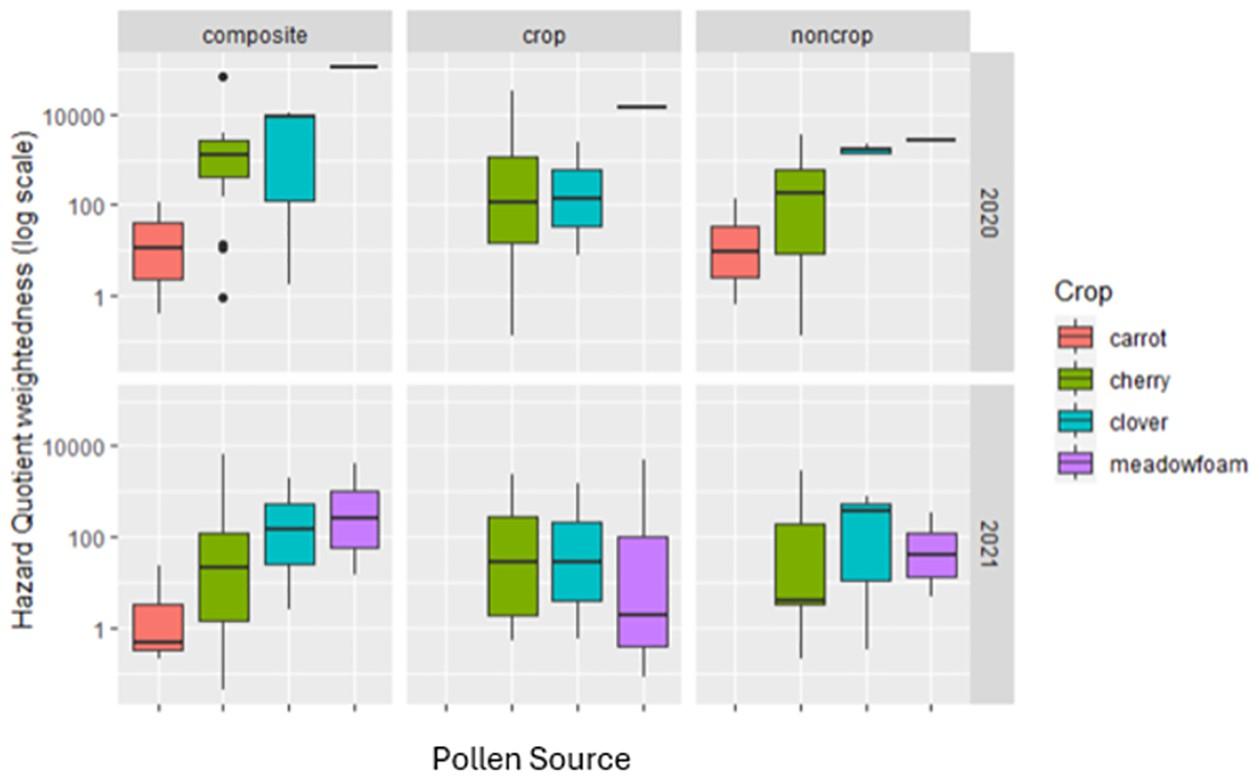
A box and whisker plot of the Index of HQ weightedness for each pollen source within composite and color sorted subsamples. The horizontal line indicates the median value (Acute Toxic Load), the boundaries of the are the 25 and 75^th^ percentiles and whiskers represent the most extreme datapoints that is no more than 1.5 times the length of the box.

HQ weighted by the mass of pollen collected at peak bloom varied across crops. HQ for total pollen from colonies placed in meadowfoam increased and in carrot decreased relative to the other crops when accounting for mass of pollen collected (Fig 4), with HQ for the latter being 200- and 10,000-fold higher compared to carrot seed (for 2020 and 2021, respectively).

**Fig 5:**
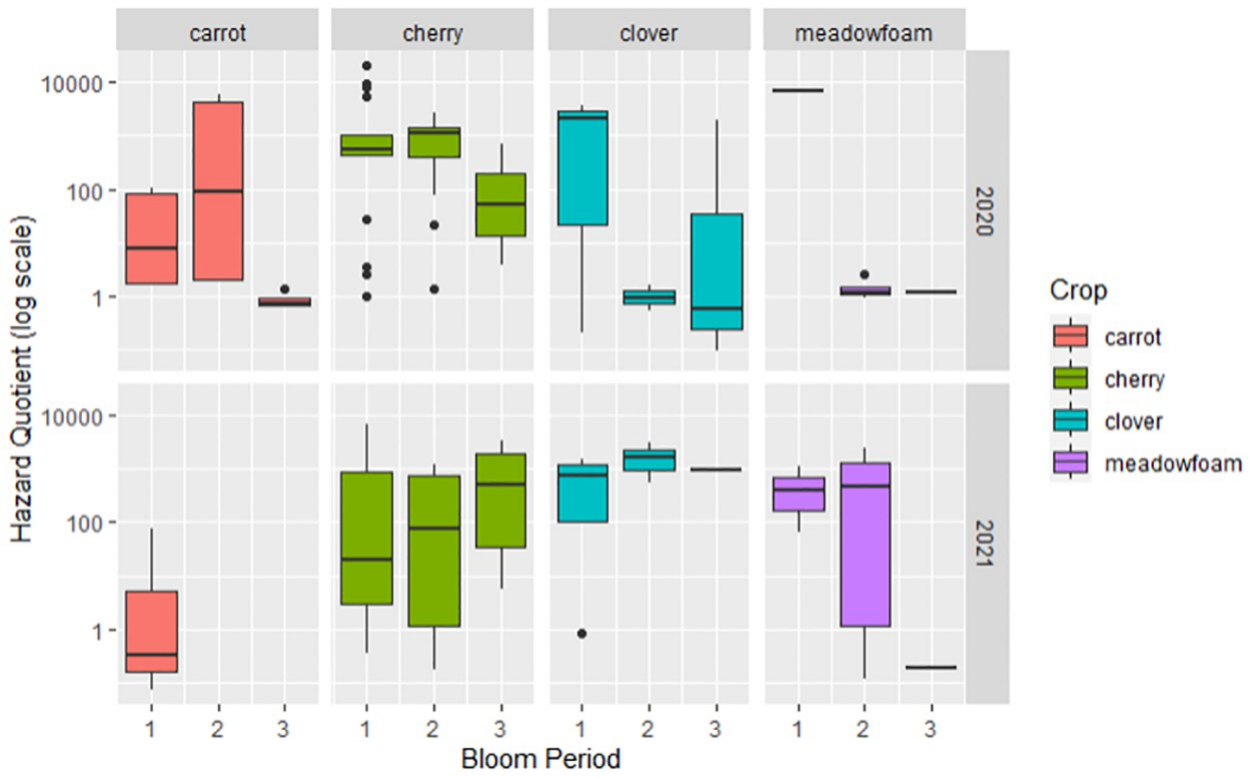
A boxplot showing the HQ of composite samples over time, by bloom period. The horizontal line indicates the median value (mass); the boundaries of the box are the 25^th^ and 75^th^ percentiles. The whiskers represent the most extreme datapoints that are no more than 1.5 times the length of the box.

To determine if time was related to HQ value, the best fit for the data was a combined GLMM and a zero-inflated binomial model. The dataset was highly skewed and we used a zero-inflated model to determine if there was a separate process effecting zero values. Pollen samples from some sites had consistently low mass over time, meaning that some sites were more likely to have no detectable HQ value associated with them. Therefore, the combined model accounts for both processes that can be causing zero values and processes which influence HQ as a continuous variable. We found that bloom period was not significantly related to transformed HQ values and did not improve the model’s fit (Table 3, Supplementary Information). Site, pollen type, and crop system all influenced if pesticides were detected within a sample or not as random effects. That is, these factors influenced if pesticides were detected at all in a sample (HQ = 0 or HQ ≠ 0). Bloom period and day of the year (1–365) affected the HQ values if HQ was non-zero. That is, these factors influenced the value of HQ when pesticides were detected. HQ values changed over time, but were not associated with a particular trend (Fig 5).

**Table 3:** Regression results from the general linear mixed effects model. This table displays the results of the linear model and the binomial model, the value associated with each variable and the p-value. The AIC for this model is 3338.5 and BIC is 3384.8 with 482 degrees of freedom on the residuals. The factors of Site and Crop contain 39 and 4 groups respectively; *n =* 493.

	**Effect Type**	**Variable**	**Variance**	**Sd**		
**Conditional Model**	Random	Site (Intercept)	0.06375	0.2525		
		Crop (Intercept)	0.22950	0.4791		
**Zero-inflated model**		Site (intercept)	2.5635	1.6011		
		Pollen Type (intercept)	0.1599	0.3998		
		Crop (Intercept)	2.3943	1.5474		
	**Effect Type**	**Variable**	**Estimate**	**SE**	**z-value**	**p-value**
**Conditional model**	Fixed	Intercept	4.671042	0.897346	5.205	1.94e-7
		Bloom period	0.014872	0.057645	0.258	0.796
		Day of the year	-1.011292	0.005873	-1.923	0.0545
**Zero-Inflated Model**	Fixed	Intercept	-1.261490	0.935159	-1.349	0.177
		Mass of pollen type	-0.002587	0.002587	-1.607	0.108

## Discussion

We describe the first effort to detect the power of sampling pollen as proxy for changes in pesticide hazard from agricultural cropping systems at state level. Pesticides were detected in the majority of samples, and HQ values were frequently above commonly reported thresholds of concern [28,31], especially within cherry systems. We found that power to detect a modest change in pesticide hazard (5% across a five-year period) varied considerably by the crop surveyed, with the lowest number of sampling sites being 190 sites (at a cost of $177,080) for clover (using 2021 sampling data) to 7,194 sites (at a cost of $3.35 million) for meadowfoam (2021 sampling data). The potential for detecting modest changes in pesticide hazard is grounded in the historic changes in bee toxic load over the past two decades; counties in the pacific northwest experienced a maximum change of 5% from the late 1990s to the mid-2010s [39]. Our study determines the number of sites needed to detect a 5% change every year for five years, however the value of HQ detectable by each sampling scheme was not consistent. For example, if sampling cherry in 2020, it would take 278 sampling events to determine differences between years with a minimum HQ value of 575. However, if sampling meadowfoam in 2021, it would take 7194 sampling events and the detectable difference in HQ would be one third of the cherry samples (HQ = 154). The interpretation of the results was complicated by the fact that in some cases almost half of pesticide detections were from products not registered on the crop being pollinated. The levels of quantification (LOQ) for each pesticide fluctuated from sample to sample as mass was not consistent. This allowed for the detections of pesticides in the color-sorted subsamples which were not found in the composite samples. Moreover, pollen trapped from colonies pollinating carrot seed was heavily weighted to non-crop pollen, a situation which was less pronounced in the other three cropping systems. The problem of hazard was also complicated by the fact that the total mass of pollen collected by honey bees at peak bloom varied across crops. As a result, the total daily exposure to pesticides to the colony (a product of the mass of pollen collected and HQ value of the pollen) resulted in further separation between colonies with the highest pollen collection (meadowfoam) as compared to the colonies with the lowest collection (carrot seed). Notably, however, the time of sampling was an important factor in understanding changes HQ as values changed with the day of the year and the bloom period of the crop. Overall, our findings suggest that post-registration monitoring of pesticides by state agencies may be feasible, but difficult to interpret.

Through our study of the variation in HQ within and across crops we were able to estimate the costs of monitoring for pesticides in pollen with sufficient power to detect a 5% change in HQ for the state of Oregon would cost between $129,548 to $3.35 million. To help contextualize the magnitude of sampling that would be needed, it is helpful to consider the number of colonies used to pollinate these crops in the state, as well as the area dedicated to these crops. For example, Oregon grows approximately 12,000 acres of sweet cherry each year in the Mid-Columbia and Willamette Valleys [60], which means finding sites for every ~86 to ~48 acres of cherry production. However, this becomes much more difficult in crops like meadowfoam which comprise only 4,000 acres in the state and would require samples to be taken every ~1 to 1.3 acres of meadowfoam production [61]. Moreover, some of these crops are pollinated by relatively small numbers of colonies, which would require sampling every colony used for pollination in crops such as meadowfoam (2,584 colonies are estimated to be contracted to pollinate meadowfoam [62]) and it would still be deficient by 528 to 1,013 colonies. Increasing the percent change in HQ over a five year period would help lower the total number of samples need, although are decision to use finer rates of change are based on previous recommendations [63]. Cost, however, may ultimately drive efforts to implement a sampling system sufficiently powerful to detect these changes. Currently, the state of Oregon spends approximately $750,000 on water quality monitoring annually [64], however, the range in cost for detecting differences in HQ was wide ($2,410,152 in 2020 and $175,216 in 2021). Water quality monitoring is also ranked as the most important environmental quality to stakeholders, [65], above pollinator protection.

Hazard Quotient estimates from the total pollen (composite) collected by bees significantly differed across crops in only one year of our study (2020). HQ estimates from pollen collected from colonies contracted to cherry pollination being twice those of colonies contracted to meadowfoam. However, this trend did not hold in 2021. Furthermore, pesticides which are approved for use within the focal crop system and active ingredients which do not appear on approved labels were frequently found within all pollen types. By separating pollen into color-sorted subsamples, we also found pesticides that were not detected within the composite samples. It is common to detect pesticides in bee collected pollen [23,24,26,36,66–68], and so the frequency of detection is not surprising. Although it was not possible to trace the source of pesticides detected in color sorted sub-samples, it should be noted that drift [69–72] rather than the use of a pesticide not registered for the pollinated crop is likely. Furthermore, our methodology of sorting pollen which has been stored in a freezer could potentially allow time for diffusion of pesticides across the lipid-rich pollen pellets [57,73].

By examining the incoming mass of pollen to the hive, we were able to highlight the problem with estimates of HQ when considering the quantity of pollen being collected by colony from a landscape. HQ is a unitless value which is connected to the LD_50_ of individual adult honey bees [21,31]. However, as Thompson points out [29], estimates of HQ based on pesticide detection in pollen do not account for the consumption rate of that pollen by different castes in a honey bee colony. Overlooked in this critique of the application of HQ to understanding the risk of pesticide residues in pollen to honey bee colonies, is the fact that some pollen is collected at exceptionally low quantities, while others are collected in such large quantities that it is not immediately consumed, but accumulated in the comb in reserve. The implications of this imbalance on risk to the colony have not been previously explored, adding to the gap in our knowledge of how exposure to pesticides in pollen manifests itself in meaningful endpoints [46,74]. Unlike the EPA’s Risk Quotient (RQ), which estimates the amount of pollen reaching each individual bee, HQ does not estimate how much pollen is consumed, and thresholds which imply critical levels of individual bee hazard are largely unvalidated and understudied [30–32]. This leads to an issue with current reliance on HQ: RQ and HQ are often not aligned [29] and while HQ in its many forms dominates the literature, it does not have strong validation for the impacts on individual bees or consequences for colonies [28]. This study highlights one potential underlying reason that HQ may not be connected to colony endpoints. The extent of this problem is best illustrated in our study by the difference between cherry and meadowfoam HQ when the total mass of pollen entering the colony at peak bloom is considered. On the basis of traditional HQ calculations, colonies contracted to cherry pollination face the highest risk from pesticide contamination in pollen. Colonies in meadowfoam, however, yielded 1.9 to 3.4 times the amount of pollen. Consequently, when we weight HQ by the increased pollen intake in meadowfoam, we are no longer able to detect a separation in HQ between these two crops. The nearly double HQ value of cherry over meadowfoam, is eroded by the nearly double intake of pollen in meadowfoam. Our findings of the differential intake of pollen may help contextualize other studies that have documented that floral resources outside of the focal crop contribute to pesticide hazard through increased contamination [20,24,26,27,75,76] or to dilution with clean pollen [77,78]. By taking into consideration the mass of pollen brought into the colony when calculating HQ a shortcoming with a reliance on this metric can be brought into focus. What is neglected by weighting HQ by the mass of incoming pollen is the rate and duration of the consumption of contaminated pollen by the colony. More than anything our findings draw attention to the fact that the current practice of estimating HQ from trapped pollen may not fully capture the exposure of pesticides to individuals in the hive, which may explain why the metric is poorly linked to hive endpoints [31].

We found that temporal variation is an important factor when sampling for HQ. The model of best fit was a zero-inflated model as, like many biological datasets [54,79], there were many points where the response variable (HQ) was zero. Pollen identity, pollen mass, site, and cropping system all influenced if HQ was likely to be zero. That is, some sites (and therefore also the crop associated with that site) had HQ values of zero more frequently. The GLMM was then modeled in a negative binomial distribution, and the final model of best fit included both the day of the year (1–365) and the bloom state of the crop. Taken together, this demonstrates that both the focal crop and the surrounding landscape are influencing the HQ value at the pollen trap. This is consistent with other studies which find that landscape context matters to HQ values [22,23,26,58,77,80]. This is particularly pronounced in a heterogeneous landscape like that of Oregon [40,41]; bees have the opportunity to collect from a wide variety of available pollen. The difference between pesticide hazard within the foraging area of the bees and the crop of interest may be greater in heterogenous landscapes than compared to regions which rely on a few key monocrop products. This variation in HQ values could be due to several factors. Due to the episodic nature of pesticide exposure, pesticide contamination in pollen is easily missed. This could weaken the connection between pesticide residues and crop-specific pesticide use practices. Shifting from a bloom-state focused pollen sampling strategy (i.e. during peak bloom) to monitoring which documents changes in HQ over long periods of time [23,81] may be the most effective. Adding landscape use within the foraging radius of the honey bee colony would be likely to improve the fit of the model; however, these type of models are not in use by states seeking to monitor pesticides.

Honey bees are highly studied and relatively easy to transport throughout a region, while also already being incorporated into pesticide risk assessment processes. Leveraging their usefulness as an agricultural pollinator to collect information about pesticide contamination and the health of other bees within a landscape is promising. However, despite their generalist foraging behavior, caution should be used honey bee collected pollen to monitor for changes in pesticide residues over time. Bee species with smaller or larger foraging radii, for example mason bees (*Osmia)* or bumble bees (*Bombus)* may be better suited to capture field to field variation [82,83], but these may also distort the hazard patterns experienced by honey bees. There are other potential ways to reduce costs of monitoring schemes and increase predictive power. There could be merits, for example, to limiting the number of pesticides tested within a sample. By nature of their high LD_50_ values, insecticides contribute disproportionately to the HQ value and focusing on just these chemistries could reduce testing costs. Traynor et al (2016) also found that high fungicide HQ contributions were associated with hive loss and queen events over the course of the pollination circuit in monitored hives [31]. Incident reporting on bee poisonings and associated HQ values could be one way to validate currently common thresholds, as has been done with application-rate HQ estimations [84].

## Conclusion

In current monitoring schemes, honey bees are used as ecological sensors, collecting pollen from within a foraging radius and enabling testing at the hive, much in the same way that water quality monitoring is used to assess stream health. Such monitoring plays an important role in ground-truthing voluntary actions (like MP3s and BMPs) as well the assessment of risk by pesticide regulators. However, the connection between pesticide contaminated pollen collected by bees and the actual risks of these pesticides to a honey bee colony is more complicated than the connection between the water sample and aquatic insect health. In aquatic systems, pesticides are applied to fields and then wash into surface water which can be collected at specific points [85]. The pesticides within the water can be assessed for their impacts on the health of stream organisms [86,87], chemical residues can be related to application rates of chemicals [88], and the overall state of the watershed determined. Finally, different streams and sites can be targeted for restoration and mitigation efforts [70,89–91] based on the results of sampling.

While other studies have noted the limitations of HQ metrics [28,29,31] we expand this criticism by highlighting a number of challenges that may make estimates of HQ from trapped pollen samples less informative than water quality samples. First, connecting honey bee collected pollen to pesticide practices in a cropping system are more nuanced compared to water quality sampling. Pollen is not collected evenly from the surrounding floral resources [92,93] and pesticide contamination from the focal crop pollen may be amplified or diluted. As in numerous other studies [25–27,94,95], we found that the amount of focal crop pollen in a pollen trap was not always a large proportion of the total pollen collected by the colony. This represents a key challenge for using pollen sampling as a post-registration monitoring tool, namely the inability to reliably link pesticide contaminating pollen back to a pesticide use practices in a specific crop where honey bees are being contracted for pollination.

A second challenge that we discovered was the high variation in HQ within sites, across sites, within a crop, across time periods within a crop and across crops. We estimated that the high variability required relatively high sample effort to detect interannual changes in hazards that are above those that have been experienced historically. To this end, it is likely that existing state-level pesticide monitoring conducted by may be well below the levels to detect meaningful changes.

Finally, we demonstrated that HQ values from trapped pollen do not currently account the fact that the mass of incoming pollen can vary widely across pollen trapping periods. This difference in pollen mass can lead to HQ estimations representing distortions in the pesticide exposure of the colony. The incoming pesticide load may differ, even when HQ values are equivalent.

In spite of the challenges we found in using pollen sampling as post-registration tool, such sampling may prove useful in characterizing risk when pesticides are being registered or reviewed. EPA employs full scale field studies (Tier III) when risk cannot be characterized by either lab studies on individual bees (Tier I) or on studies where bees are forced to forage on plants that have been treated with the pesticide (Tier II). Tier III studies, however, often are expensive, costing an estimated $1.2 million, compared to Tier II ($75,000–150,000) or Tier I ($30,000–50,000) [96]. Estimates of spatiotemporal variation of HQ from studies such as ours, could help regulators model field variability to supplement Tier III studies to refine estimations of risk. A major challenge, however, remains in interpreting HQ values from trapped pollen in terms of risk. Although we demonstrated that the importance of accounting for the mass of pollen collected by bees in calculating HQ, this represents part of the picture of exposure, as it does not account for the rate in which pollen is being consumed by different bee castes or pollen stored within the colony and consumed over time [46,74]. More research needs to be done to better characterize how contaminated pollen translates to actual exposure of bees to the pesticides. Such work may resolve the issue of establishing meaningful thresholds for HQ levels, which are commonly used [21,31] but appear at odds when compared to models of pollen consumption, such as BeeREX [29], which are used in risk assessment.

## Supporting information

S1 File Synergistic Pesticide Labs Summary of pollen testing methodology. (PDF)

S2 File A table displaying every detection of pesticide from the study as an individual row in the dataset. (CSV)

S3 File A table displaying the total HQ values and summary information for each SampleID. (CSV)

S4 File R code for creating all of the figures. (RMD)

S5 File Reporting limits from Synergistic Pesticide Labs that includes the batch-specific level of detection, levels of quantification, and the 292 pesticides each sample was tested for. (XLSX)

S6 File Pesticide test information on the empirical formula, isotopic mass, MS polarity, MS ion, retention time, and precursor for pesticide tests performed. (XLSX)

S7 File Power analysis on untransformed HQ data. (DOCX)

S8 File Metadata. (DOCX)
