# Supplementary material for: The power to (detect) change: Can honey bee collected pollen be used to monitor pesticide residues in the landscape?
Source: PLoS One. 2024 Sep 26;19(9):e0309236. doi: 10.1371/journal.pone.0309236 (PMC11426543; doi:10.1371/journal.pone.0309236)
Supplement: S1 File — (PDF) [file pone.0309236.s001.pdf]

I. Introduction & Summary

This report describes the extraction and analysis of the Multiresidue method for over 300 pesticides in pollen.

The QuEChERS extraction is an internationally accepted extraction method developed by Anastassiades in 2002. The extraction used for these samples is the European Standard EN 15662 which is unique for employing citrate salts to buffer pH. Pollen is a challenging matrix with various coextracted materials, requiring a GCB dSPE cleanup that results in the reduced recovery of some planar pesticides. Analysis was done via LC/MS/MS and GC/MS/MS.

II. Materials & Methods

A. Equipment

- GC/MS/MS – Thermo TSQ 8000 EVO GC/MS/MS, Thermo Trace 1310 GC, Autosampler AS 1310
- LC/MS/MS – Thermo TSQ Endura LC/MS/MS, Vanquish Binary Pump H, Vanquish Autosampler – Split Sampler H, Vanquish Column Compartment
- Balance – Sartorius Top Loading Balance ED2202S
- Barnstead EASYpure II RF Water Filter, Model D7031
- Centaur Bottletop Dispenser
- Beckman Coulter Centrifuge, Model Allegra-6R, Cat 366816, Ser ALR99J4S

B. Materials

- Acetonitrile (ACN), ChemProducts, C-A0142-60
- QuEChERS Packet EN15662, Phenomenex, AH0-9041
- dSPE, GCB/PSA/$MgSO_4$, Phenomenex, KS0-9517

C. Standards

All working standards used for spiking quality control samples, building calibration curves, and CCVs were current for use with available CoAs on file.

III. Procedure

A. Sample Receipt/Preparation

Organic Bee Pollen sample material RD20 008 was verified to be free of residues prior to analysis and used for matrix spike quality control samples. Pollen samples were tested as provided in entirety.

B. Project Batching

Up to 20 samples were grouped in a batch with the following Quality Control samples:

- Method Blank (MB)
- 2 spiked replicates (MS/MSD) at a mid-concentration (10x the LOQ)

C. Extraction

Samples were tested by the buffered QuEChERS method EN 15662 followed by cleanup with a GCB dSPE:

- Weighed pollen samples in a provided 50mL centrifuge tube.

- Added 10 mL DI H_2O to all tubes.
- Spiked MS/MSD.
- Added ACN to all samples to bring to a final volume of 5 mL. Shook briefly to mix contents.
- Added QuEChERS packet: 4 g $MgSO_4$, 1 g NaCl, 1 g TCD, 0.5 g DHS. Shook vigorously for 1 minute and centrifuged 5 minutes at 3000 RPM.
- Took aliquot of supernatant, transfered to GCB dSPE cleanup tubes. Shook vigorously for 2 minutes and centrifuged five minutes at 3500 RPM.
- Took aliquot of supernatant for analysis.

D. Analysis

All samples were analyzed by LC/MS/MS and GC/MS/MS, with a calibration for each run.

Calibration curves were prepared in matrix extract with Multiresidue Working Standard Mixes and fitted to a linear curve consisting of 6 points, ranging from 10 to 500 ng/mL at the low calibrant. CCV injections at the mid-curve concentration bracketed all samples.

The results were calculated using the following equations:

- Reporting Limits:

$$\text{Reporting Limit (ppm,}\frac{\mu g}{g}) = \frac{(\text{LOQ,}\mu g/mL)(\text{Final Volume,mL})(\text{Dilution Factor})}{(\text{Initial Mass,g})}$$

- Sample Detections:

$$\text{Amount Detected (ppm,}\frac{\mu g}{g}) = \frac{(\text{Raw result,}\mu g/mL)(\text{Final Volume,mL})(\text{Dilution Factor})}{(\text{Initial Mass,g})}$$

- Spiked Samples:

$$\%\ \text{Recovered} = \frac{(\text{Raw result,}\mu g/mL)}{(\text{Expected result,}\mu g/mL}$$

- CCV:

$$\%\ \text{Drift} = \frac{(\text{Detected Standard Concentration} - \text{Expected Standard Concentration})}{(\text{Expected Standard Concentration})}$$

IV. Validation Parameters & Acceptance Criteria

A. Accuracy can be described as a measure of exactness of an analytical method and is displayed as the percent recovery of an added amount of analyte.

B. Precision is the closeness of agreement among individual test results from repeated analyses of samples. Repeatability, or intra-assay precision, generates results over a short time interval under identical conditions and is reported as percent relative standard deviation (%RSD).

C. Instrument **linearity** and **range** must be defined. Linear calibrations must have at least 5 concentrations across the expected range of sample detections with coefficient of determination, $r^2 \geq 0.98$. CCVs must be within 30% drift of initial calibration.

D. Acceptance Criteria Guidelines:

**Calibration Curve**	$r^2 \geq 0.98$
**MB**	Non detect
**UTC (Native)**	If expected to be control, any detection should be examined for potential crossover; if presence of analyte unknown, any detection should be compared to spiked validation sample detections. Legitimate detections in the native *within the calibration curve* should be subtracted from spiked matrix samples. Legitimate detections in the native *above the calibration curve* result in the rejection of study results with that matrix sample.
**MS Replicates**	70–130% mean recovery preferable, but may be outside that range with precision ≤ 20% for each spike concentration. Control limits may be established for analytes in a specific matrix.
**CCV**	% Drift ≤ 30%

V. Results

Please refer to the associated spreadsheets for data, reporting limits, and summaries.

Where analytical detection were confirmed by retention time and acceptable mass spectra ion ratios, residues were reportable between 50% of the Reporting Limit to the Reporting Limit with qualification.

Limited pollen sample amounts often limit the amount of extract generated, therefore diluting extracts to bring residues above the calibration curve on scale is often not possible. Values detected above the curve are considered estimates and qualified on reporting.

VI. Definitions

- CCV – Continuing Calibration Verification
- CoA – Certificate of Analysis
- DHS – disodium hydrogen citrate sesquihydrate
- DI - Deionized
- dSPE – Dispersive Solid Phase Extraction
- GC/MS/MS – Gas chromatography with tandem Mass Spectrometry
- GCB – Graphatized Carbon Black
- LC/MS/MS – Liquid chromatography with tandem Mass Spectrometry
- LCS – Laboratory Control Samples
- LOQ – Limit of Quantitation
- MB – Method Blank
- $MgSO_4$ – Magnesium Sulfate
- MR – Multi-Residue
- MS – Matrix Spike
- TCD – trisodium citrate dihydrate
