# Supplementary material for: The power to (detect) change: Can honey bee collected pollen be used to monitor pesticide residues in the landscape?
Source: PLoS One. 2024 Sep 26;19(9):e0309236. doi: 10.1371/journal.pone.0309236 (PMC11426543; doi:10.1371/journal.pone.0309236)
Supplement: S7 File — (DOCX) [file pone.0309236.s007.docx]

| **Sampling Scheme** | **Year** | ***n***  **Sites** | **Number of tests^a^** | **Cost (USD)** |
| --- | --- | --- | --- | --- |
| Cherry | 2020 | 190 | 380 | $167,760 |
| Carrot |  | 449 | 898 | $418,468 |
| Clover |  | 404 | 808 | $376,528 |
| Meadowfoam |  | 1842 | 3,684 | $1,716,466 |
| Any crop, all peak |  | 1470 | 2,940 | $1,370,040 |
| Cherry | 2021 | 43 | 86 | $40,076 |
| Carrot |  | N/A | N/A | N/A |
| Clover |  | 938,944,115 | 1,877,888,230 | $87,509,315,180 |
| Meadowfoam |  | 764 | 1,528 | $712,048 |
| Any crop, all peak |  | 1,855 | 3,710 | $1,728,860 |

*a* – The number of tests was twice the number of sites, to reflect that the sampling was performed across two different years.

A table displaying the results of the power analysis (n) and cost analysis in USD of sampling schemes. The value *n* represents the number of sites needed for each year that sampling occurs; the number of tests represents 2*n^a^,* the required sampling effort for both sampling years. The results show how many composite samples would need to be taken to detect a 5% change in HQ values.
