# Supplementary material for: The power to (detect) change: Can honey bee collected pollen be used to monitor pesticide residues in the landscape?
Source: PLoS One. 2024 Sep 26;19(9):e0309236. doi: 10.1371/journal.pone.0309236 (PMC11426543; doi:10.1371/journal.pone.0309236)
Supplement: S8 File — (DOCX) [file pone.0309236.s008.docx]

Meta-data

S1 – Synergistic Pesticide Labs Summary of pollen testing methodology

S2 – A table displaying every detection of pesticide from the study as an individual row in the dataset (new_total3)

- SampleID: The total pollen sample
- Date: The end date of collection
- Year
- Site: The name of the site
- SampleType: The sample classification (composite or color group number)
- Mass_perc: the percent mass (note: for composite samples, this is the same as the absolute mass)
- Crop: Cropping system the sample was collected from
- Crop_or_NonCrop: the pollen identity
- Mass_abs: The absolute mass of the pollen sample (calculated from the percent and total mass of the sample)
- Pesticide: The name of the pesticide detected
- Detection: The detection (ppm)
- Type: Fungicide, Insecticide, or Herbicide (F, I, H)
- LD50: The acute oral LD50 of the pesticide
- Source: The source of the LD50 value listed
- app_clover: Is the pesticide approved for use in clover
- app_meadowfoam: is the pesticide approved for use in meadowfoam
- app_carrot: is the pesticide approved for use in carrot
- app_cherry: is the pesticide approved for use in cherry
- Indv_HQ: Hazard quotient of the individual detection, calculated from the detection and the LD50
- Pest_Count: The number of pesticides found within the same sample
- SumHQ: The total sum of all HQ values found within the sample
- Transf_HQ: Log(HQ+1)
- Approved: Is the pesticide approved for use in the system it was detected in?

S3 – A table displaying the total HQ values and summary information for each SampleID

- SampleID: The total pollen sample
- Date: The end date of collection
- Year
- Site: The name of the site
- SampleType: The sample classification (composite or color group number)
- Mass_perc: the percent mass (note: for composite samples, this is the same as the absolute mass)
- Crop: Cropping system the sample was collected from
- Crop_or_NonCrop: the pollen identity
- Mass_abs: The absolute mass of the pollen sample (calculated from the percent and total mass of the sample)
- Pest_Count: The number of pesticides found within the same sample
- SumHQ: The total sum of all HQ values found within the sample
- Transf_HQ: Log(HQ+1)
- Bloom_rank: the bloom state of the crop

S4 – R code for creating all of the figures

S5 – Reporting limits from Synergistic Pesticide Labs that includes the batch-specific level of detection and the 292 pesticides each sample was tested for

S6 – Pesticide test information on the empirical formula, isotopic mass, MS polarity, MS ion, retention time, and precursor for pesticide tests performed

S7 – Metadata
